# Nanoinformatics for environmental health and biomedicine

**DOI:** 10.3762/bjnano.6.253

**Published:** 2015-12-21

**Authors:** Rong Liu, Yoram Cohen

**Affiliations:** 1 UC Center for Environmental Implications of Nanotechnology, University of California, Los Angeles, Los Angeles, California 90095, United States; 2 UCLA Institute of the Environment and Sustainability, Los Angeles, California 90095, United States,; 3 UCLA Chemical and Biomolecular Engineering Department, Los Angeles, California 90095, United States

**Keywords:** nanoinformatics

Nanotechnology has become a significant enabling technology for a wide array of industries being integrated across diverse areas such as medicine, electronics, biomaterials, and energy production. For example, nano-scaled systems have been designed and utilized for safe and effective targeted delivery of therapeutic agents, demonstrating the rapid advancements of nanotechnology in medical-treatment and diagnosis. At the same time, there is also mounting concern regarding the potential impact of nanotechnology on the environment and human health. As a result, there is a global drive to ensure that the development of beneficial nanotechnologies is accomplished in a responsible manner so as to avoid adverse impacts on environmental and human health.

In order to develop safe-by-design nanomaterials for their various intended applications, large amounts of data are being generated for better understanding and mapping the toxicology and pharmacology of nanomaterials. Nanomaterials data are typically sought regarding their physicochemical and structural properties, environmentally related properties, toxicity behavior, processing information, production levels, environmental releases, and more. Accordingly advanced informatics techniques are urgently required for the collection and curation, management (e.g., achieving and sharing), analysis and modeling of the large amount of data involved with nanotechnology processes and materials (i.e., “nano-data”). In order to address these requirements, nanoinformatics has emerged over the last decade as “The science and practice of determining which information is relevant to the nanoscale science and engineering community, and then developing and implementing effective mechanisms for collecting, validating, storing, sharing, analyzing, modeling, and applying that information.” [1]. At present, nanoinformatics focuses primarily on: nano-data management and database development, nano-data curation, assessment of the value of information in nano-data, literature mining for nano-data collection and meta-analysis, data mining/machine learning of nano-data (e.g., development of quantitative structure–activity relationships (QSARs)), simulation of the fate and transport of nanomaterials, nano-bio interactions, and assessment of potential environmental and health risks associated with nanomaterials.

As an interdisciplinary field consisting mainly of nanotechnology and data science, nanoinformatics has significantly advanced over the last decade, playing an increasingly important role in research and development in nanomedicine and environmental health impact assessment of nanomaterials (often termed NanoEHS). In addition, efforts in nanoinformatics research have provided in a multitude of tools and resources that are being made available through nanoinformatics cyberinfrastructures and web platforms (e.g., nanoinfo.org [2] in the US and eNanoMapper [3] in the EU). However, much of the current research and advances in nanoinformatics are not documented in dedicated resources and, given the interdisciplinary nature of nanoinformatics, are dispersed throughout a wide range of sources and journals. As a consequence, researchers and practitioners in other fields of nanotechnology have been at a disadvantage not having easy access to the most recent resources and tools provided by the nanoinformatics research community. Accordingly, this Thematic Series is devoted to bring together the state-of-the-art in nanoinformatics with a particular focus on the latest related developments/applications for environmental health and biomedicine.

In this Thematic Series, recent advances in the development of databases are reported. These databases represent a collection of valuable data related to the physicochemical properties and bioactivity of nanomaterials. In one contribution, the latest version of caNanoLab is described along with a critical discussion of the challenges associated with database development for nanomaterials, as well as the needs for nano-data curation and sharing by the biomedical research community [4]. The latest development of the eNanoMapper database for nanomaterial safety information is summarized in another contribution [5], while a third contribution reports on the NanoE-Tox database that is concerned with the ecotoxicity of nanomaterials [6]. In addition, important improvements are reported for the Nanotechnology Consumer Products Inventory that progressively documents the marketing and distribution of nano-enabled products into the commercial marketplace [7].

The progress in nano-data curation is covered in two contributions. One describes the Nanomaterial Data Curation Initiative, a collaborative effort by the nanoinformatics research community for nano-data discovery and extraction, quality assessment, integration, and reuse [8]. Another contribution illustrates key concepts, and discusses current practices and challenges in the field of nano-data curation [9]. In order to facilitate nano-data discovery and extraction, a data collection framework was developed [10] through ISA-TAB-Nano (a set of standardized specifications for nano-data representation). Advances in automating nano-data discovery and extraction is the subject of two other contributions that report on using advanced literature/text mining techniques, such as natural language processing [11] and corpus-based automatic information extraction [12]. In addition, bibliometric and social network analysis is introduced and adopted in the field of nanoinformatics to identify collaboration networks and developmental patterns of nano-enabled drug delivery for brain cancer [13].

As an imported aspect of nanoinformatics, recent advances in data mining/machine learning of nano-data are also reported in this Thematic Series. In one study, the toxicity of ZnO nanoparticles to zebrafish (measured by mortality rate (%)) was correlated to two principal components calculated from nanoparticle size and surface properties using Kriging estimations [14]. Another contribution reports on the development of models to predict the cytotoxicity of PAMAM dendrimers using molecular descriptors [15]. Nanomaterials that have potential to cause disease (e.g., TiO_2_ nanoparticles, carbon black, and carbon nanotubes) were also identified using biclustering of gene expression data and gene set enrichment analysis methods [16]. Various visual analytical approaches (e.g., bipartite graphs, log-ratio analysis, and multidimensional scaling) are demonstrated in another study for exploring the impact of manufactured nanoparticles (ZnO and TiO_2_) on soil bacterial communities [17], which is an area of nanoinformatics that is only now receiving increased attention.

The present Thematic Series also presents a simulation tool for estimating the release and environmental distribution of nanomaterials, which provides critical information for the environmental impact assessment of nanomaterials [18]. Another contribution addresses the issue of nanomaterial risk assessment and proposes a decision analysis scheme for furthering nanoinformatics work [19]. This work considers an array of decision analysis techniques (e.g., multicriteria decision analysis, value of information, weight of evidence, and portfolio decision analysis) that are potentially capable of assessing and classifying the multitude of available nanomaterial data. Such an approach can serve as the basis for both establich a decision making process and future research priorities in the field.

This Thematic Series was made possible by the contribution of numerous authors to whom we owe our gratitude. We appreciate the time and effort of the numerous referees that helped shape this Thematic Series and we are also grateful for the unwavering support of the team at the Beilstein-Institut. We particularly acknowledge and commend the Beilstein Journal of Nanotechnology for its open access policy, which has provided a wonderful incentive for researchers and practitioners to contribute to this journal while is freely available to all scientific and professional communities.

Rong Liu and Yoram Cohen

Los Angeles, October 2015
